# On Puerperal Insanity

**Published:** 1859-01-01

**Authors:** 


### Art. II.—ON PUERPERAL INSANITY.

We have selected the comprehensive title of "Puerperal Insanity," as the heading to some observations on unsoundness of mind especially attached to the puerperal state, in preference to that of puerperal *mania*, which, although much more frequently used, is certainly imperfect; inasmuch as mania is only one of the various forms assumed by the intellectual disorder, which so frequently supervenes during this period. In the outset we would state that the period to which we here allude as the "puerperal," extends, in our plan, from the moment of conception to about a fortnight after the cessation of lactation; or, in the absence of this latter function, to about two months or ten weeks after delivery. And this period admits of a natural division into three minor ones, during which the mental disorder is characterized by phenomena and reactions sufficiently marked—viz., (1) the period of pregnancy; (2) that of delivery and six weeks succeeding; and (3) that of lactation and a week or ten days subsequently. During each of these sub-periods mental disorder may supervene, due to the physiological and quasi-pathological condition; and may assume the forms of mania, melancholia, hallucinations, illusions, and partial delirium (or monomania). These forms are generally admitted by writers; we venture to add to them *acute dementia*—an illustration of which we shall give from our own note-book, in due course. Dementia, in a chronic form, has been allowed by some authors to form a certain proportion of the cases.

In attempting to give a view, as comprehensive as our limits will permit, of the present state of our knowledge of this difficult section of morbid psychology, we propose in some measure to follow the plan of M. Marcé, a recent French writer on this subject, whose contributions have been for some years appearing from time to time in the Continental journals; and who has now collected them, with additions, into one compendious treatise.* We shall avail ourselves largely of his valuable collection of data,

* Traité de la Folie des Femmes Enceintes, des nouvelles accouchées, et des nourices; et considérations medico-legales qui se rattachent à ce sujet. Par le Dr. L. V. Marcé. Paris, 1858.

and add such observations from other sources as may seem desirable for further illustration.

M. Esquirol remarks (in 1819) that although much had been written concerning the diseases to which women recently confined were liable, and the consequences of the supposed *metastasis* of the lacteal secretion, yet very little had been said on the subject of the *mental* maladies which make their appearance after delivery, or during lactation; and in a brief memoir he treats upon the frequency, the causes, the nature, and the treatment, of these affections. For long after this, however, writers passed hastily over the subject, only enumerating the puerperal state as one frequent cause of mental unsoundness. We have before us one systematic work on insanity, of no very ancient date, in which the whole of this branch of our science is passed over in seven or eight lines.

And yet it would appear that puerperal insanity was well worthy of separate study, whether from the frequency of its occurrence, the gravity of its character, its clearly-marked etiological relations, its amenability to treatment, or lastly, its important legal bearings.

There are many reasons why it is difficult to determine with any approach to accuracy the absolute number, or the relative proportions, of these diseases to the number of persons confined. In private obstetric practice such cases are not generally made known; the friends of the sufferer naturally wish it to be kept secret; and the medical attendant in the majority of cases has no interest in keeping the statistics of his practice with any accuracy. It would appear that in institutions devoted to lying-in women, there would be no difficulty in arriving at some definite conclusions; but here the case is even worse, for instead of having no data, we get incorrect ones—from this cause, that in such places, the women generally only remain about a fortnight after delivery; whereas insanity may occur a month or six weeks after this period; and, where the child is suckled, it may occur at any period during lactation. Hence arises the discrepancy of the accounts, and the generally small stated proportions of such cases to the whole number of deliveries. Dr. Reid stated in this journal, in 1848, that out of 3500 women confined at the General Lying-in Hospital in Westminster, where the patients remained three weeks after delivery, only nine were attacked with insanity. At Queen Charlotte's Lying-in Hospital, in 2000 cases there were 11 of insanity; this greater proportion was considered due to the greater number of unmarried females admitted here. In two other collections of cases we meet with 950 in which no instance of insanity, and 1888 in which only one occurred, which was very soon cured.

When we inquire into the proportion which puerperal insanity bears to the other forms, we are enabled to arrive at some definite results. M. Esquirol states, in the memoir before mentioned, that at the Salpetrière from one-twelfth to one-tenth of the females there received are affected with puerperal insanity. In 1119 women there were 92 such cases; in 1812 and 1813 there were 60 in 600 cases. In private practice the proportion is still larger, the same authority giving the numbers are 21 in 144 cases. At Bethlehem, during five years, there were 111 cases of puerperal insanity out of the entire number of 899 women admitted. Other statistics are as follows—the periods of admission not specified :—

| | Entire Number of Females admitted. | Cases of Puerperal Insanity. |
|---|---|---|
| Hanwell ... ... ... | 703 ... ... | 79 |
| Reported by Dr. Macdonald | 691 ... ... | 49 |
| By M. Parchappe ... ... | 596 ... ... | 33 |
| By M. Leller ... ... | 97 ... ... | 11 |
| By Dr. Webster ... ... | 282 ... ... | 17 |
| By Dr. Kirkbride ... ... | 2752 ... ... | 116 |
| By M. Mitivié ... ... | 242 ... ... | 9 |

Summing up these, and many other details, we find that about one-twelfth part of the women who are received as insane into asylums, are so in consequence of the puerperal condition, or as a sequel to it.

It is next proper to inquire at what period of the puerperal state these cases occur. Bearing in mind the division into sub-periods above noticed, we find that by far the greater number of cases occur in the second, or that immediately after delivery, and in the next six weeks. In the 92 cases mentioned by Esquirol, 54 occurred in this period, and 38 during lactation. In a report from Hanwell, in this journal, in 1848, 43 cases were mentioned, of which 4 occurred in the first period (pregnancy), 26 in the second, and 13 in the third, or suckling period. Dr. Macdonald found, in 66 cases, 4 in the first period, 44 in the second, and 18 in the third. M. Marcé, in 79 cases, found 18 developed during pregnancy, 41 after confinement, and 20 during lactation. M. Marcé has collected 310 cases from various authors, which are thus distributed :—

| | |
|---|---|
| Developed during pregnancy ... ... ... | 27 |
| After confinement ... ... ... ... ... | 180 |
| During lactation ... ... ... ... ... | 103 |
| Total | 310 |

In forming a judgment upon these statistics it must not be forgotten that the absolute number of women confined is much larger than that of those who suckle. The influence of lactation, therefore, is not fully shown by such relative proportions as the above.

After these preliminary considerations we shall proceed to offer some observations upon these cases of insanity in chronological order; and first upon those occurring during pregnancy. During 30 or 35 years of female life, the uterus and its functions exercise a most powerful and important influence, not only over the animal economy, but also over the moral and intellectual nature. This influence is very specially marked during pregnancy, when the entire nervous system frequently acquires an excessive mobility and sensitiveness to all impressions—evidenced by vomitings, syncopes, cramps, and vertigo; *occasionally* partial paralysis, deafness, amaurosis, chorea, epilepsy, and hysterical crisis. The moral nature indicates its subjection to the same influence by an unwonted excitement of the faculties, or more frequently by a tendency to extreme depression and discouragement. This is in many cases purely psychical, but not in all. In primiparæ especially, there are many physical causes—such as the important changes in life, residence, occupations, &c.; in these, also, pregnancy is not always a happy event; but, even amongst those who have every cause for rejoicing, the same depression will often occur, and one fixed idea will often take possession of the mind. There is a strong conviction of a fatal termination, or there is a fear of some deformity in the child, &c. At the same time the digestive functions are disordered, the respiration and circulation are troubled, sleep is broken and disturbed, and there is a tendency to fainting. On the other hand, some naturally melancholic women present a strange excitement and gaiety during this period. The singular tastes and propensities manifested at such times are too familiar to require notice; but some of the recorded cases are sufficiently curious to merit quotation. Vandermonde (quoted by M. Marcé) relates the case of a woman who became hydrophobic during the first four months of each of her eleven pregnancies. Soon after conception she began to drink very little, and by-and-bye the horror of fluids was so great, that she could neither drink herself, nor could she bear others to drink in her presence. To cross a running stream was almost impossible to her. M. Cazeux relates the case of a young lady, *primipara,* who, during her pregnancy, felt the most unconquerable aversion to her husband, whom at other times she most tenderly loved; and of another, who took so decided an antipathy to her home, that she had to go into the country to be confined, in spite of all the efforts of her reason.

All these peculiarities are not insanity; they are moreover marked by one favourable characteristic—viz., that they, for the most part, diminish or disappear as pregnancy advances, and *very rarely* last after delivery, or pass into insanity at that period. M. Marcé states that out of 79 cases of disturbed intellect during labour, he only found six who had exhibited any well-marked peculiarities during pregnancy. This furnishes an excellent index for treatment. To correct by the mildest means any marked somatic disorder, to prescribe moderate exercise, and a careful moral hygiene, are the principal points to be kept in view—always remembering that, in a short time, nature will in all probability effect its cure, if not too officiously interfered with, or disturbed by so-called remedies too potent for the occasion.

But these intellectual disturbances may pass into veritable insanity; and between the one form and the other there are innumerable gradations, of which it is often difficult to say to which category they belong. M. Marcé has some remarks on this point, which are valuable:—

"There is one point which has struck us, and which, we believe, may aid the physician in his diagnosis; it is, that the tendency to sadness, and all the modifications of character and intelligence with which we meet at the outset of pregnancy, become, in general, less and less marked as this advances, and especially after passing the third month. Now, we observe exactly the contrary in the facts of true mental alienation. Setting aside those cases, where conception gives immediately the signal for the outbreak of insanity, this does not generally appear until after the third month—more frequently the sixth or seventh; and it does not in general disappear during pregnancy at all."

Two observations, by the same writer, are worthy of notice, in reference to the *causes* of the insanity of pregnant women; the first is that, after *heritage*, moral influences of a painful character are amongst the most frequent causes—such as circumstances requiring a concealment (were it possible) of the pregnancy. The second is, that this form of insanity is not so frequent amongst primiparæ as amongst those who have had two or three children. At the same time he states that the number of his own observations is too small to make this a well-ascertained conclusion.

A previous attack seems to predispose to a recurrence. Sometimes the attacks occur with alternate pregnancies; some are only observed with male children; and many other varieties are noticeable. The precise time of the outbreak is variable; sometimes it is truly and strictly *sympathetic*;* the appearance of the mental disorder is synchronous with conception, and its disappearance with delivery. At other (and more frequent) times the

* The word "*sympathetic*," as here made use of, requires a little definition, being intended to imply something more specific than its usual significance. We call one affection *sympathetic* with another, when it is strictly coincident with it as to commencement and termination; when the two appear clearly to stand in the relation of cause and effect; and *sublatâ causâ, tollitur effectus*. Thus, a lady ex

*sympathy* is imperfect, and the disorder appears from the third to the seventh month.

The form assumed by the mental disorder is in the majority of cases *melancholy*, with tendency, it may be, to suicide, with or without hallucinations. In other cases the form is maniacal. It may be stated here that whatever is the special type of insanity of puerperal women, occurring at any period, whether mania, melancholia, partial delusions, or dementia, these differ in no respect from the corresponding form occurring in the non-puerperal condition. Dr. Gooch remarks, that "if a physician was taken into the chamber of a patient, whose mind had become deranged from lying-in or nursing, he could not tell, from the mere condition of the mind, that the disease had originated in these causes." M. Marcé says also: "Disons seulement que rien dans les symptomes, la marche et la physionomie de ces deux maladies (mania and melancholia) chez la femme enceinte, ne permet de la distinguer des maladies de même nature observées dans des conditions ordinaires de santé." Dr. Macdonald,* however, considers that there is one sign which attaches especially to puerperal insanity:—"In the acute form of the mania which succeeds parturition we observe an intensity of mental excitement, an excessive incoherence, a degree of fever, and, above all, a disposition to mingle obscene words with the broken sentences—things which are rarely noted under other circumstances. It is true that in mania modest women use words which in health are never permitted to issue from their lips; but, in puerperal insanity, this is so common an occurrence, and is done in so gross a manner, that it early struck me as being characteristic." Dr. Campbell also remarks, that the patient, "though remarkably devout when sane, now launches out into such a torrent of obscene language, that one would be astonished that respectable females could have become familiar with such expressions." These peculiarities belong more especially to the form of mania which succeeds confinement.

The prognosis of these cases of true derangement is very uncertain. It is unfortunately not the case that they terminate periences at each menstrual period violent pains in the face and neck; a fibrous tumour of the uterus is detected and removed, and the neuralgia disappears entirely. Esquirol attended a young lady who became deranged on a suppression of the menses, and was restored immediately they began to flow again. Guislain relates the case of a girl who had prolapsus of the uterus, and who was affected with the most profound sadness, with tendency to suicide, whenever the neck of the uterus presented itself at the opening of the vagina. Such cases as these are illustrations of what we call complete sympathy: those in which the mental affection lasts much beyond the organic disease, or physical cause, or where it ceases whilst such causes are still in operation, are examples of *imperfect* sympathy. Cases of the former class are much more favourable as to prognosis and indications of treatment than the latter.

* "Journal of Psychological Medicine," No. 3, p. 534-5.

with delivery in more than about one-third of the number. In M. Marcé's 19 cases 7 terminated at this period, or near it; but 4 of these cases were purely *sympathetic*—that is, had begun at the period of conception; such as these may generally be expected to terminate thus favourably. It is rare that an attack of insanity begins and ends within one pregnancy. M. Esquirol relates one case, and Madame Boivin another; besides these, M. Marcé has not met with any.* In the majority of cases the labour seems to exert little influence upon the march of the disorder; some cases remain permanently incurable; others continue for months and even years, without much change, and then gradually subside. The termination by death is not frequent. This fact is of much practical importance, as throwing discredit upon the plan that has been occasionally adopted, of attempting to cut short the mental affection, by inducing premature labour. This can only be expected to succeed when the affection is of that strictly sympathetic nature above noticed. The treatment must be conducted on principles precisely similar to those which guide us in analogous cases, uncomplicated by pregnancy; being careful to avoid energetic measures. Indeed, in many cases, it must be almost entirely expectant; comprising such precautions as are necessary for the safety of the life of mother and child.

During labour a transitory delirium occasionally supervenes, which, although not frequent, it is important to notice from its legal relations. At the latter period of labour, when the pain becomes most intolerable, some women become so excited as to lay violent hands upon the child, if possible—to attempt suicide—and to view with hatred the husband and child to whom they are most tenderly attached. Osiander delivered of twins a woman, whom two strong men could scarcely prevent from throwing herself out of the window. He also relates the case of a plethoric woman at Strasbourg, who, in the midst of the most violent pains, demanded that she should be cut open, and herself obtained a knife to attempt it. Another instance, by the same writer, would appear to have something incredible in it. It is that of a negress, who, being seized with delirium during a long and painful labour, cut open her body, extracted the infant, and recovered! We shall have occasion to return to this subject, should our limits permit us to speak of the judicial relations of pregnancy and parturition: we now pass on to notice the insanity of the recently-confined and of nurses; and first concerning its etiology.

The earliest notice of this is found in Hippocrates, who announces in the 40th Aphorism of the 4th section, that "a congestion of blood in the mammæ announces insanity." Some

* Since writing the above, we have met with a few instances, mentioned by various authors.

writers have quoted cases in support of this opinion. Van Rossum relates that M. Picters saw a woman yielding blood instead of milk; the fourth day she became maniacal, and died the seventh. Planchou relates a similar phenomenon;—on the fourth day of the flow of blood, loquacity supervened, and a maniacal delirium followed which lasted till death, a month afterwards. But cases of this kind are few in number; and others of a totally opposite significance abound, in which blood flowed from the breast, without any evil consequences; and also in which insanity appeared without any such symptoms. It would not tend to any valuable practical result to enter minutely into the ancient theories of the causes of the mental disorders of puerperal women. They are all founded upon the hypothesis, that after labour, the milk escapes by the breasts and by the lochial discharge, and that when from any cause this secretion is turned from its natural direction it affects the brain, either by being deposited upon or within it, or by making the blood impure. Thus, even for such men as Sydenham, Levret, and Van Swieten, the suppression of the milk or of the lochiæ is an essential condition for the development of the mental disorder. What are the facts which bear upon this view?—

Examining the reports of such cases as occurred during the six weeks succeeding labour, *in those who did not suckle,* we find that a considerable proportion did not occur until after the first month—that is, until a period when the lochial discharge has naturally subsided. Amongst those which occurred earlier we find eleven illustrative cases. In one, the suppression of the discharge coincided with the outbreak of the delirium; in two the suppression was subsequent to it. In seven the lochiæ continued to flow three weeks or a month, being at most slightly diminished at the period of the appearance of the mental disturbance. Lastly, in one case, the patient had attacks of insanity after repeated confinements, and in some the lochiæ stopped, and in others continued to flow. M. Marcé, commenting on these facts, proposes to reverse the usual verdict, and to say that "puerperal insanity in some cases suspends the lochial secretion, by reason of the general perturbation of the system which it induces; but more frequently exerts no influence upon it."

If we examine also the phenomena attendant upon the secretion of milk, in reference to the development of the psychical disturbance, we shall find that the relation is equally undefined. Sometimes the insanity breaks out at the very time when the secretion of milk commences, and at other times at the period of weaning; moreover, in many cases, the secretion continues unaffected during the whole course of the most marked derangement. We may conclude, therefore, that the proximate cause of puerperal insanity is not to be found in the suppression of milk, or of the lochiæ.

In rejecting this theory we naturally wish to fall back upon some other; and we attribute the development of puerperal insanity to the reactions between a system predisposed to such derangements, and the normal physiological conditions which are found after confinement: just as in constitutions predisposed to tetanus, or nervous delirium, these will be developed after the slightest accidents or operations. The pains of labour, the lively emotions which often accompany it, and the large suppurating surface which results after the expulsion of the fœtus, will bear a very close comparison with the course and results of a serious surgical operation. After a time, also, temporary secretions are established, which, both at their commencement and their termination, necessarily induce serious changes both in the circulating and nervous systems. Dr. Gooch's opinion on this subject is worthy of much attention:—

"The cause of puerperal mania is that peculiar state of the sexual system which occurs after delivery."

He afterwards explained and commented upon this as follows:—

"What I meant was this: the sexual system in women is a set of organs which are in action only during half the natural life of the individual; and even during this half they are in action only at intervals. During these intervals of action they diffuse an unusual excitement throughout the nervous system—witness the hysteric affections of puberty, the nervous susceptibility which occurs during every menstrual period, the nervous affections of breeding, and the nervous susceptibility of lying-in women. I do not mean that these appearances are to be observed in every instance of puberty, menstruation, pregnancy, and childbirth; but that they occur sufficiently often to show that these states are liable to produce those conditions of the nervous system. . . . . Dr. Marshall Hall thinks that the susceptibility of the puerperal state is to be explained by mere exhaustion; and does not at all depend on the influence of anything specific in the condition of the several organs at that time; but would an equal or a greater degree of exhaustion at *any* time occasion the disease? This is a question of fact, that I should answer in the negative. I have seen patients who have been deranged in childbed, and who had recovered, at a future period much more exhausted by illness, and much more agitated in mind, without the slightest appearance of mental derangement."

Having a condition of body at least as well prepared for delirium as it would be after a serious operation; and a state of mind in general much more so; we cannot feel any surprise at derangement supervening on certain occasions, especially when the *predisposing causes* are powerful.

At the head of these, as is the case in all mental affections, we may place hereditary influence. M. Esquirol states the proportion of those thus liable, to those attacked, at about 2 in 5. Dr. Gooch says: "A very large proportion occurred in patients in whose families disordered minds had already appeared." Dr. Burrows says that out of 80 women, who became delirous after labour, above half had an hereditary predisposition to insanity. The following are other statistics :—

| | No. of cases of puerperal insanity. | | No. of cases of hereditary predisposition. |
|---|---|---|---|
| By Dr. Helfft, of Berlin | 131 | — | 51 |
| „ M. Weill, of Stephansfeld | 30 | — | 14 |
| „ M. Marcé | 56 | — | 24 |

No doubt these proportions would require considerable increase, as there are great numbers of cases where no history can be got; there are also numbers which are allied to families where great nervous excitability prevails, perhaps not amounting to alienation, but liable to be developed into that under any favourable conditions.

Anæmia, whether primitive or consecutive to repeated pregnancies or hæmorrhages, is a powerful predisposing cause of mental alienation. Its symptoms are here, as in ordinary cases, paleness of skin and of mucous membrane, small pulse, weakness of the digestive functions, emaciation, and weakness of the muscular system; the *bruit de souffle* is likewise heard if the anæmia passes certain limits.

Authors differ as to the effect of repeated pregnancies upon the production of mental alienation; it has generally been considered that the liability is greater in the first pregnancy. M. Marcé disputes this, however, and finds amongst 57 patients only 14 primiparæ; and amongst the 43 remaining cases 13 had been confined five, six, and even nine times. This he explains by the greater debility produced by repeated confinements.

The influence of age is sufficiently marked: the further removed is the patient from the most favourable age for childbearing, 20 to 30, the greater is the liability to mental disturbance, judging from the statistics. These are of rather too complicated a nature to be made clear in a brief statement.

A previous attack appears strongly to predispose to a recurrence of the mental disorder; or an attack of insanity in the non-puerperal state has very much the same influence. It appears also, according to some authorities, that the attacks in successive confinements do not increase in intensity—the patient often recovers from the later ones more readily than from the earlier. Esquirol relates the case of one woman who was attacked by puerperal insanity ten times; and on the last occasion recovered in a few weeks. Dr. Ramsbotham considers that the chances of a patient becoming insane after a subsequent confinement, who has been so in one, are very small, "Although (he adds) as the former attack proves that a predisposition then existed, and may still be operating, that very circumstance would strongly impress us with the *possibility* of a recurrence, and would induce us sedulously to avoid every exciting cause, and to use the utmost degree of care for its prevention, not only in the next, but all the following labours." Dr. Gooch appears to hold the same opinion; he says—"I have attended many patients who came to town to be confined, because they had been deranged after their former lying-in in the country, and, excepting Case No. I., not one of these patients had a return of their disease." Dr. Montgomery relates a case of a lady who became insane after eight successive confinements; and several others in a less marked degree illustrative of the same position. Our opinion is that one attack strongly predisposes to another; and that, if the instances of successive attacks are not so numerous as might be expected, it is because the care and pains alluded to by Dr. Ramsbotham are not without their result in preventing them. In some few cases it has been observed that the *sex* of the infant acted as a predisposing cause—women having got over the confinement of a girl with safety, after repeatedly having suffered when the child was a male.

Of the occasional or exciting causes of puerperal insanity we are not well informed. It does not appear that difficult labours exercise much influence in the majority of cases; nor are many of the recorded cases connected with extreme hæmorrhage. We cannot speak definitely as to the relation which it bears to eclampsia; Merriman, Gooch, Esquirol, Frias, Selade, Billod, and Dr. Reid, each relate one instance of such apparent dependence.

In this Journal, for the year 1850, Dr. Webster relates some cases in which he attributes to chloroform the production of the mental disorder; Dr. Simpson relates facts having a very opposed significance. The question is one of very great importance, but one which must be decided by prolonged experience.

Moral emotions will, no doubt, exercise a strong influence upon the development of mental alienation; the cases in which the affection is distinctly traceable to these are certainly not very numerous; but the number would be very much augmented had we full histories of each case. In women who do not nurse, the first menstruation has a strong influence upon the development of this affection: in 60 cases mentioned by M. Marcé eleven became insane exactly at this period. It is worthy of notice also that, amongst those who do nurse, a not inconsiderable number begin to be insane about the same time—*i.e.*, the sixth week. Out of 22 cases six were of this nature. It is scarcely necessary to point out the practical bearing of this observation; nor how necessary at such a period is the most careful physical and moral hygiene. Errors in diet, exposure to cold,* imprudences of various kinds, inflammation and suppuration of the mammæ—all these have been observed to act as exciting causes. We have ourselves observed one case which in the outset was clearly dependent upon a sudden pleurisy. In this, as in other diseases, however, etiology is an obscure science; for the most part the affection will not be due to any one cause, but to the action of many upon a constitution enfeebled or otherwise predisposed.

These general observations apply to the insanity of the newly-confined, and to that of nurses indiscriminately; it will now be convenient, following the plan of M. Marcé, to separate the two forms, and make some remarks upon each.

The forms of mental unsoundness enumerated by this author as occurring shortly after confinement are mania, melancholy, partial lesions of the intelligence, hallucinations, intellectual and instinctive monomania, and "a special variety of mental enfeeblement, which seems to be caused by excessive losses of blood, and is easily cured by appropriate treatment." These forms of alienation are not equally frequent; amongst M. Esquirol's 92 cases 49 were mania, 35 melancholy, and 8 chronic dementia. In the 44 related by M. Marcé 29 were mania, 10 melancholy, 5 partial lesions, and 2, cases of temporary intellectual enfeeblement. It is worthy of notice that in the insanity of nurses the proportions between mania and melancholy are much more equal than those here noticed.

In the form of puerperal insanity now under notice, the cases almost all begin at one of two periods; either in the first eight or ten days after confinement, or towards the fifth or six week. M. Marcé remarks that in his 44 cases, 33 commenced in the first ten days, and 11 about the sixth week. We do not find in other authors the same precision of classification.†

* M. Esquirol lays great stress upon cold as an exciting cause. He says:—'Le refroidissement, l'impression du froid, de quelque manière qu'ils aient lieu, sont de tous les écarts les plus à redouter ; l'exposition a l'air frais, l'application de l'eau froide, soit que l'accouchée s'expose à des courants d'air froid, ou qu'elle marche en plein air, soit que l'accouchée ou la nourice plonge ses membres dans l'eau froide, la coupe des cheveux, l'abus des medicaments chauds, en supprimant les lochies, provoquent la folie. Chez nos 92 aliénées, quatorze fois l'aliénation mentale a été provoquée par des causes physiques, et dans ces quatorze cas, dix fois l'impression du froid a causé la maladie."

† M. Esquirol's 92 cases commenced as follows :—

| | | | |
|---|---|---|---|
| 16 | became delirious | from | the 1st to the 4th day. |
| 21 | ,, | ,, | 5th ,, 15th ,, |
| 17 | ,, | ,, | 16th ,, 60th ,, |
| 19 | ,, | ,, | 60th day to the 12th month. |
| 19 | ,, | | after forced or voluntary weaning. |

These 92 cases include both forms of puerperal insanity—that of the newly-confined, and that of nurses.

It is not necessary to enter minutely into the phenomena of puerperal *mania,* so familiar to all of extensive obstetric experience. The outbreak may be quite abrupt, as in the case related by Dr. Reid, where the patient having fallen asleep in good health, awoke suddenly, crying out that her child was dead, and became maniacal from that moment. In the great majority of cases, however, the accession is gradual; the first change noticed is often in the eye, which assumes an expression easily recognisable, but difficult of description. The countenance is restless, anxious, and troubled; sometimes flushed and sometimes unnaturally pale. There is great excitement of the special senses; slight sounds distress the ear, light affects the eyes; there are often hallucinations. The temper changes completely, and family affection is apparently changed into the bitterest hatred; and this is particularly observed as regards the child, which the mother often attempts to destroy. Then succeeds or accompanies these symptoms the outbreak of violent delirium, with the characteristics before mentioned. At the commencement there is not always fever, and if the pulse be accelerated, it is often from the violence of the excitement alone; but after a time it becomes very rapid (especially in those cases which will be fatal); the head is sometimes hotter than usual, although the general temperature is not much raised; there is almost complete insomnia; the tongue is foul, the urine scanty, the bowels often constipated; the breath is offensive, and the skin emits an unpleasant odour. The condition of the milk and the lochiæ has been before noticed. These signs of constitutional disturbance, in the majority of favourable cases, subside long before the mental disorder.

It is of much importance to inquire whether during pregnancy or labour there are any symptoms which may lead us to apprehend insanity afterwards, and so enable us to take every precaution to guard against it. Esquirol states that it is sometimes announced by sinister presentiments even during pregnancy; but such presentiments are so frequent, and are in so small a proportion of cases followed by any evil consequences, that we cannot found much upon them. Dr. Burrows has some important remarks on this point:—

> "Puerperal delirium consequent on labour is sometimes predicted, though not absolutely developed during gestation. If, while pregnant, there be frequent hysterical affections, preternatural sensibility, unaccountable exuberance or depression of spirits, morbid aptitude to exaggerate every trivial occurrence, and attach to it great importance, suspicion, irritability, or febrile excitation; or, what is still more indicative, a soporous state, with very quick pulse—then the supervention of delirium on labour may be dreaded."

Dr. Ramsbotham adds to this, that "if a great loss of memory be present, such a result is eminently foreboded."

The characteristics of puerperal mania are so well marked, that it is scarcely likely to be confounded with any other disease. From the low muttering delirium of fever, the history will sufficiently distinguish it, as well as its own peculiar characters. From phrenitis it is sometimes not so easy; and cases will occasionally occur where the affection seems to partake of both characters. The delirium of phrenitis is preceded by headache, fever, tinnitus aurium, and flushed cheeks; the pulse is quick and sharp; all these are generally wanting in mania. In the former, the eyes are injected; not usually in the latter. The inflammatory fever of phrenitis has a character almost altogether wanting in mania. It is of the highest importance to distinguish between the two affections; since the active depletory measures required for phrenitis would be ruinous in mania.

Puerperal mania terminates by recovery, by incurability, or by death; the first appears to be much the most frequent; the last is rare. "It used to be the prevalent opinion (says Dr. Ramsbotham) that puerperal mania *never* resulted in a fatal termination. Even the late Dr. Baillie, observant as he was of disease, and well-informed upon the morbid conditions of the body in all their forms, when consulted about a case of this kind, remarked, 'that the question was not whether the patient was to recover, because of that he had no doubt, but how long the disease was to last!'—she died within a week after this opinion was uttered. Of Esquirol's 92 cases, 6 died, 1 after six months, 1 after a year, 2 after eighteen months, 1 after three years, and 1 after five years after delivery. These statistics, however, as well as those of Dr. Webster and Dr. Burrows, include not mania only, but all the forms of puerperal insanity.* In 24 cases of mania, M. Marcé enumerates 16 recoveries, 2 incurables, 2 in which after one year there was no amendment, and 4 cases of death; 1 in twenty-six days, 1 in nineteen, 1 in sixteen, and 1 in seven days. Almost all deaths in this disease result from a complication with, or transition into, *acute delirium*, the *délire aigu* of French writers. Where the insanity has been developed during the first fifteen days after confinement, it may be thus complicated from the first, but it may also occur after the mental affection has lasted many weeks. M. Marcé's sketch of this affection is graphic:—

"The agitation augments from day to day, the tongue becomes dry, the digestive functions are impeded; the pulse becomes rapid, more than 120 per minute; the face is flushed, the head hot, and the eye haggard; the skin is covered with a viscous sweat; the patient is a prey to incessant hallucinations, and exhausts herself by violent agitation and

* As this is the case with all the statistics yet published on the subject, we shall recur to the various terminations of puerperal insanity, after reviewing the different varieties.

an unceasing loquacity; she is no longer conscious of anything that is present; under the influence of her delirious ideas, or even of a veritable hydrophobia, she rejects all aliments, especially drink, and spits almost perpetually. M. Baillarger has remarked the expectoration of large yellowish *crachats*, which are unaccompanied by cough, or by any pulmonary symptoms."

Then follows a quasi-typhoid state, with fœtid breath, and still incessant spitting; the urine and fæces are passed involuntarily; insomnia is constant, and the powers are speedily exhausted—the termination being often accelerated by diarrhœa. The signs of amendment are, that the pulse becomes somewhat slower, and has more volume; the tongue moistens, the agitation is a little calmed, and the patient returns either to reason, or to the normal condition of the previous insanity. But when once the condition above described is fully developed, there is but very slight hope of any amendment; the prognosis is most grave. With regard to the prognosis of puerperal mania in general, the quickness of the pulse is the most serious sign—not that quickness which is brought on merely by agitation, and is transitory, but a continuous permanent acceleration, which does not subside even during moments of calm. The acute delirium would seem to be merely the maniacal agitation, carried to its extreme limits; and the constitutional disturbance is merely a consequence. It is of importance to recognise the beginning of this acute delirium; and *fever* is the distinctive sign between it and the ordinary maniacal agitation. Meningitis offers striking relations occasionally to this acute delirium; but in this we meet early with partial paralyses, contractions, or strabismus; the head is thrown backward, there is subsultus, coma, and resolution of the muscular fibres. Those patients who do not die of this affection are generally carried off either by diarrhœa, or by some pulmonary disease, if the insanity terminates fatally.

The pathology of puerperal insanity is as obscure as that of mental affections generally; morbid changes are found occasionally after death, of various kinds, but none that are constant or special; and it must always be remembered that *death* has taken place, for which some physical cause must exist, which is not *necessarily* connected with the psychical disorder. Esquirol has examined the bodies of patients who have died of puerperal insanity, in which there was no morbid change to be detected. Others have found thickening, or eburnation of the skull, induration, or softening of the brain, opacity and adhesions of the membranes, purulent or serous deposits, &c. &c. In one of our own patients the most careful examination failed to detect any morbid condition. Dr. Gooch relates a case which was exactly similar. As a general rule, in those cases which are fatal, whether by reason of acute delirium, or of some intercurrent malady, the lesions found in the brain are insignificant, and altogether insufficient to furnish a plausible account of the phenomena. Those which have been complicated by meningitis or cerebritis will present the usual morbid appearances; but to the disease itself there is no special morbid anatomy attached. We quote M. Marcé's conclusions on this point in full:—

"I do not wish to conceal the little importance which I attach to the results of pathological anatomy in this disease: the details which I have given are the latest data of science, but I am convinced that in a short time another order of ideas will arise concerning the researches necessary to be made, in order to *try* to ascertain the organic cause of the three or four elementary and distinct forms which constitute mental alienation. What is it that we now study when we examine the bodies of maniacs, monomaniacs, or melancholics? The more or less intense colour of the white or grey matter, the abundance of the sub-arachnoid fluid, the state of the membranes, the consistence and general aspect of the cerebral pulp; and the examination is made by means of *approximative appreciations purely personal*, without exact means of verification. But these incompletely interrogated elements constitute but a small part of the diseased organ. The state of the nerve-tubes, of the intermediate substance, the induration or ramollissement of the organ, the quantity of water which it contains, the state of the chemical elements which enter into its composition; these are all points which we should examine by the most rigorous methods which science affords; and, so long as so complex and extensive a labour is unperformed, no one has a right to say that we are ignorant (qy? *must be* ignorant) of the intimate nature and cause of insanity. *The immaterial soul cannot be diseased;* the brain then is responsible for the intellectual disturbance. Moreover, when I see forms of delirium so clearly characterised as mania, monomania, and melancholy, in their typical forms, I cannot believe that one and the same elementary lesion presides over alienations so distinct, and I am disposed to admit something special in each case."

The *treatment* is that of mania, in the non-puerperal state. As a general rule bleeding is utterly inadmissible, unless there are such inflammatory complications as would render it necessary, were there no mental aberration. The heat of head, so often observed, indicates the propriety of the application of ice to the shaved head; and blisters are often of very great service. The extremities are often cold, and must be stimulated by hot water, or mustard, and the usual obvious methods. The stomach and alimentary canal must be regulated, if necessary, by emetics and purgatives; and, after this, opium is of the greatest utility. Dr. Gooch advises hyoscyamus, and camphor, and is generally of opinion that narcotics are the most valuable remedies in this disease. The intellect is often obviously clearer from the rest obtained by their use. In the chronic stage stimulants and tonics are often required; and ammonia is amongst the most useful. Dr. Pritchard mentions oil of turpentine, in drachm doses, three times a day, as one of the best stimulants, when the stomach will bear it. The diet must be farinaceous, with a good allowance of milk, so long as the febrile symptoms prohibit animal food; in the chronic stage it must be of a free and generous order, including a certain amount of wine, or malt liquor. The rules as to isolation must be as strictly observed in this form of mania as under ordinary conditions. If it cannot be accomplished perfectly at home, it must be by removal to an asylum, as soon as the special circumstances of the case admit of it. The general management is that of ordinary mania.

Such is an outline of our ordinary English practice; some other methods have obtained reputation on the Continent, which we may briefly sketch, as they appear to have been attended with success. Tartarized antimony has been recommended by Dr. Weisener, as reducing the energy of the nervous system, without affecting the general strength so seriously as depletory measures. Dr. Elsoener relates a case, in which the patient was attacked with mania five days after delivery; he gave tartarized antimony at first in fractional doses, and ultimately to the amount of six or eight grains in the day, applying cold to the head at the same time; the cure was completed in twelve days. There might, however, be practical difficulties in carrying out this plan.

Prolonged tepid baths, for two or three hours, or more, are stated to have produced the happiest results in these cases, on the authority of M. Brierre de Boismont. It will be remembered that, some time ago, we gave a sketch of M. Pinel's method of treating delirium tremens—in which prolonged warm baths, for five, eight, ten, or even twenty-four hours, with constant cold effusion to the head, formed the principal feature, with or without the exhibition of opium. In the cases now under consideration it is necessary to bear in mind that the peculiar state of the patient will not bear so prolonged an exhausting process; and the time must be limited to two or three hours at the utmost.

M. Esquirol had great faith in injections of milk and sugar, three times a day, the diet being at the same time rigorously attended to. Camphor has at times been resorted to as an almost exclusive treatment, given both by the mouth and in injection. M. Marcé gives two or three cases where recovery was rapid under this plan; but he does not attach much value to it, nor yet to æther, which has occasionally been employed in drachm doses.

M. Baillarger has strongly recommended "milk diet" as a curative measure—alone, or combined with baths, purgatives, or narcotics. In giving two or three pints of milk daily the thirst is allayed, and a good deal of nutriment obtained, which is not exciting. This may be tolerated for a considerable time; but if it should begin to purge, it must be relinquished, as it is rare that the toleration of it is re-established. According to the special circumstances of each case, a selection, or combination of these methods of treatment, will be desirable. It is, perhaps, scarcely necessary to add, that if the delirium be anything more than transitory, all idea of suckling must be relinquished, as not only would it injure the mother, but the safety of the child would be materially compromised.

The *melancholia* of the recently-confined is less grave, at least so far as life is concerned, than mania; it is also less frequent at this period by more than one-half. Its history is very similar to that of melancholy in general, and its symptoms the same. The period of its outbreak is the same as that of the affection already noticed. It begins by signs of depression from the first, which may be mistaken for obstinacy or sulkiness; or it may commence by a degree of excitement almost maniacal, and only assume its special form afterwards. Hallucinations of the ear, the eye, and of taste, are very frequent; and there is often a suicidal propensity; very commonly, also, the child is the object of extreme dislike, and attempts are made to destroy it. M. Marcé says that in these cases he has repeatedly noticed an almost complete *analgesia;* the patient appeared insensible to pricking or any irritation. Hysterical convulsions, and a cataleptic state, have also been observed accompanying this affection. Almost all the patients present the symptoms of anæmia (as above described), with *bruit de soufflet* in the heart and large vessels.

The prognosis is not very grave, the greater number recover by far; the duration is from one month to six, or perhaps longer. The treatment is that of melancholia in general, modified in accordance with the special requirements of the condition. The principal indication to be kept in view is that the constitution is almost to *remake.* Iron and other tonics are of great service when the proper period arrives; at a proper interval after confinement the cold affusion may be used daily with advantage; in short, all means which will raise the powers of life. When this is accomplished, opium and other narcotics are as useful in this form as in mania, to allay excitement, and procure rest.

We have no particular observations to make upon the partial derangements of intellect in the newly-confined; they differ in no respect from those met with in the non-puerperal condition. They chiefly consist of hallucinations, impulses, religious scruples, and sometimes partial losses of memory. Some of these latter are worthy of passing notice. Capuron relates, that a young lady, after a painful confinement, experienced a severe emotion, which brought on syncope, lasting three days. On recovery she remembered nothing of having been confined; and this amnesia lasted some months. Louyer-Villermay relates a similar case: A young lady, after long disagreements and controversies with her family, married a man to whom she was much attached: after her first confinement an accident happened, accompanied by long weakness, on her recovery from which she had lost all memory of the time that had elapsed since her marriage, although she remembered exactly all the details of her previous life. Her marriage itself was forgotten, and she repulsed with every appearance of fear her husband and her child. Since then she has never been able to recal the memory of this portion of her life.

A species of chronic dementia is an occasional, but not frequent, form of puerperal insanity; sometimes it merely depends on debility, and is relieved by appropriate tonic treatment; but it is generally more grave in its significance.

In the early part of this paper we have alluded to acute dementia as occasionally occurring after confinement. It is mentioned by some writers, but we have nowhere seen any detailed description of it. A case which occurred to us, some months ago, seemed to be of this nature, and is certainly worthy of record. The patient was a woman of about 26 years of age, in her third confinement, apparently of healthy constitution. There had been nothing in the two former labours, nor in the nursing which followed, to account for the subsequent phenomena. On this occasion, all went on well for seven or eight days, when a peculiarity of manner, more than a derangement of health, awoke the anxiety of the friends. There was a little headache, and some occasional flushing of the face; the tongue was white, rather *creamy* in appearance, but there was no thirst. The milk flowed freely, and the lochiæ were natural; the sleep was not much affected. The peculiarity of manner was this: when spoken to, she appeared to understand perfectly, and to have an idea of the answer in her own mind, but on trying to express it, two or three words were uttered, and the rest were lost. Thus, on being asked if she had much pain in the head, she would say, "No, not in my ——" and then, after a pause, she would add, "I know very —— if I could only ——." When the attendants tried to explain, she would stop them, and make another attempt; and, on again failing, she would put her hand to her head, and appear trying to remember, and the face would flush with the effort. She was quite conscious that she forgot words, and seemed a little annoyed at it; but generally the expression of the face was calm, and even happy. The pulse was not above 75, and soft. But by degrees the power of comprehension diminished, and the faculty of speech was lost: there was more fever, and the patient became unconscious. The friends described something like convulsive movements of one arm; a quasi-typhoid state supervened, and on the eighth day from the commencement of the attack she died. A very careful post-mortem examination, 36 hours after death, failed to reveal any morbid change. The brain and its membranes might have passed for an entirely healthy specimen. The other organs were in the normal puerperal conditions, without evidence of inflammation, or any other morbid action. We are unable to offer any theory as to the pathology of this case.

The insanity which supervenes upon the state of lactation is closely allied to that of parturient women. The puerperal state proper is supposed to end with the flow of the lochiæ; but lactation maintains a condition which has been not inaptly termed a "prolonged puerperal state." "In virtue of the lacteal secretion the woman is so far removed from her physiological state; she is more nervous, more impressionable, and more accessible to morbific influences, which she would easily have resisted at another time" (Marcé). The mental alienation of nurses may be naturally divided into two classes; (1) those which appear within the first six weeks after labour; and (2) those which do not appear until after six, eight, twelve, or more months of suckling, or immediately after weaning. The cases of the first class are too closely allied to those already noticed to require any detailed examination. If insanity does not appear in the first six weeks, it is very rare to meet with it before the sixth month: out of 22 cases, M. Marcé states that he has only once seen it appear at any intermediate period; this was in the third month. An etiological deduction of some value may be drawn from this fact—viz., that as the great majority of these cases appear only after prolonged lactation, the cause is to be sought in the anæmia and debility produced by this; and it may be inferred that this cause is the most powerful, after heritage, and the other *general* causes of mental alienation.

In all cases of extreme debility or exhaustion, nervous accidents are to be expected in some form—palpitations, vertigo, weakness of sight, or the other senses, neuralgic pains, partial paralysis, contractions—all these, with the constitutional symptoms of imperfect nutrition, are met with in nurses in whom a too prolonged lactation has induced an anæmic condition. It is quite natural to expect, therefore, that in those who are so predisposed, by the operation of the influences before described, alienation should supervene.

But if the weakness produced by lactation be so powerful a predisposing cause of insanity, how does it happen that this so frequently comes on a few days after weaning? On a first glance we should suppose that the cessation of so abundant a secretion ought to tend to strengthen the economy; and it has sometimes certainly the effect of checking mental disturbance. M. Marcé relates the case of a woman who had five attacks of delirium after five successive labours; yet three times she tried to suckle her children. "When she suckled she was in a state of maniacal agitation, which came on a few days after labour, lasted during the whole lactation, and regularly ceased eight or ten days after weaning. When she did not suckle, the delirium ceased a few days after the milk fever." Such facts as these are not frequent, and authors justly attribute a dangerous influence to the period of weaning. But weaning is not *always* justly chargeable with the effects attributed to it: it may be that a patient has evinced nervous symptoms of various kinds, and weaning has been ordered; the insanity appears, but it was probably pending before, and the weaning has simply not checked it. From whatever cause, however, or combination of causes, it may be, a considerable proportion of cases of puerperal insanity do arise at this period. Out of 38 cases of this nature, related by Esquirol, 19 occurred just after weaning; and in 22, related by M. Marcé, six were at this period. "Enfeebled women, it appears, may nurse for a great number of months, and only fall ill at the moment when lactation is suspended; for even an exhausted organism habituates itself during some time to losses which enervate and weaken it in a regular daily manner. We see it daily in individuals subject to hæmorrhoidal discharge, or a purulent secretion from an issue; if these are suppressed suddenly they produce in the economy a reaction as dangerous in proportion as the subject is inclined to nervous accidents. One of our patients, Madame X——, had an accession of insanity after each of her confinements. When she suckled, the accession was deferred until the time of weaning, when it broke out in full force" (Marcé). But there is still another condition, and one which has a different etiological significance. The suppression of an abundant secretion may determine a state of *plethora*, to which the insanity may be due; and it is of importance to recognise this fact, and the cases which illustrate it, as the treatment will require to be essentially different.

As in the insanity of parturient women, so these cases may commence abruptly, evidently the sequel to some exciting physical or moral cause; or they may come on gradually, in which case, almost invariably, the disorder of the physical health will precede the outbreak of the mental disturbance. The precursory symptoms are those of anæmia, already mentioned; and they are of the utmost importance to mark, inasmuch as welldirected care in this prodromic period may avert the threatened aberration.

The older authors were accustomed to attribute all these mental disorders in nurses to suppression of the milk. When the delirium is very intense it does sometimes happen that the secretion ceases; but this is not frequent. In 40 cases, Dr. Macdonald only noted six in which this was the case; and M. Marcé states, that in all his cases of insanity consequent upon lactation, he has not met with one in which the secretion did not continue, often requiring special means to prevent the distention of the breasts.

It is worthy of observation, that the cases of mania and melancholia are nearly of equal frequency amongst nurses, although the causes operating in their production are chiefly depressing. There is nothing distinctive in any of the forms which insanity assumes during lactation to require special mention, after what has been said before. The prognosis is not very grave. M. Marcé enumerates 26 cases, of which 20 were cured, 1 uncured, 3 were lost sight of, and 2 dead. Of the 20 cures, 6 took place in the first five weeks, 5 from ten weeks to three months after the commencement, 2 in four months, 1 in seven months, 1 in eight months, 6 in from ten months to two years. Of the 3 who were lost sight of, 2 were notably improved, and 1 seemed to threaten dementia. The 2 who died were both melancholic; one died of phthisis; the other refused all nourishment, and so perished.

The general outline of the treatment is the same as that prescribed before for the insanity of parturient women. If the disease can be taken when the physical disorders are giving evidence of what is impending, much may be done by careful and gradual weaning, and by a strict physical and moral hygiene, to prevent the outbreak. In any case the child must be weaned, were it only for the obvious reason that it is in danger whilst in the care of an insane mother. When the secretion is stopped, we must have recourse to a decided tonic treatment—iron, quinine, cold affusions, change of air and scene, moderate exercise, and generous diet. In that class of cases which depend upon phethora, of course this treatment is inapplicable; in its place, mild evacuants, even stronger ones, may be necessary occasionally. In all other particulars the treatment must be conducted on the general principles laid down for insanity, in its ordinary forms; and what has been said before concerning isolation is here equally applicable.

The history of puerperal insanity cannot be made complete without a few illustrative cases, two or three of which we shall abstract and condense as far as possible—selecting them from sources which will be most likely to be new to most of our readers. A very instructive and interesting case is related by M. Legrand du Saulle, in the *Annales Medico-Psychologiques,* for April, 1857, in which the patient had two attacks, one of mania, and the other of melancholy; both yielded to treatment, but the patient finally succumbed to hæmoptysis and rapid phthisis.

Marguerite B—— was brought up by poor parents, in the country, in excellent principles, and had the rudiments of a moderate education; her conduct was always irreproachable. She always enjoyed good health, and menstruation was developed, and continued normally. She married happily when eighteen years old. In the three first years of her marriage she had two children, both of whom she nursed, at intervals of one year. When pregnant for the third time, her habits, tastes, and manners underwent a sudden change; she indulged in gay and obscene conversation, forgot the sentiment of modesty, sought the society of men, and when her husband remonstrated, she overwhelmed him with reproaches, and even blows. In trying to escape from the house, one night, she fell from the window, and abortion, with enormous hæmorrhage, followed. The fœtus was about five months old. After this, during her recovery, she surprised every one by her calmness, and her perfect return to her original character. But scarcely was she convalescent, when a violent mania broke forth, and on May 28th, 1849, she was removed, by order of the authorities, to the asylum of the Côte-d'Or, almost in a state of acute delirium. Under treatment by baths daily, and purgatives twice a-week, the excitement rapidly subsided, and in forty-eight days she went home perfectly well, and was as before, an excellent manager, and a quiet, staid mother of her family. She again became pregnant, without any mental affection supervening; she was confined of a little girl, whom she suckled eleven months. Scarcely had she weaned the child, when she fell into a state of emaciation; she lost her natural affections, and very soon was plunged into a state of utter depression and inertia, which necessitated her entry into the asylum again, on the 27th of March, 1851, twenty-two months after she had gone out. "At our first visit, we found Marguerite before a window, immovable and mute, with fixed eye and open mouth, without consciousness of time, place, or persons; she seemed in a sort of ecstasy, and living in an imaginary world. The sensation of hunger was not strong enough to rouse her; she did not touch anything that was given to her, and had to be fed like an infant; in a word, she was in a state of melancholy, with stupor." She had prescribed a generous diet, and tonic medicines, with alkaline and sulphureous baths. In about twenty days some amendment was perceived; her eyes seemed to recognise, even with pleasure, some objects; and at different times she even articulated intelligible words; she paid some regard to propriety, which had before been quite neglected. On the 20th of April she had a blister applied to the arm; she spoke a few reasonable words, and then broke into a long and loud laugh. April 25th: "It seems that Marguerite is awakening as from a frightful dream, and that she is endeavouring to gather together her ideas—to retrace her thoughts. She appears unquiet, *chagrin*, frightened; she looks at us with terror, and hesitates to answer." On the 1st of May she seemed to be well; on being asked what she had thought about during the long period of her immobility, she replied, "Oh, I don't know; I had always before my eyes my little child, which my husband was cooking in a vessel of boiling water. And then I heard it cry; but I was as if dead. I would have taken it off the fire, but could not, my hands and feet were so bound." There were also hallucinations of another kind. She had seen her niece taking her first communion, and, on going out of the church, a gendarme took her away to prison, where she had died. May 10th: The improvement was progressive; she still answered slowly, but rationally; her physical health was excellent. On the 25th, "we promised her that she should go home in a few days; but, on the 1st of June, on going to inform her that her discharge was made out, we found her spitting blood in large quantities." The remainder of the case is but the history of a rapid phthisis, with perpetual hæmoptysis. She died on the 2nd of July.

Autopsy, thirty hours after death:—The body appeared anæmic. The vessels of the brain were empty; the cerebral pulp was firm; its consistence was greater than natural, as if macerated in alcohol. In the chest the pleural cavities contained about ten ounces of fluid; the left lung was full of tubercle, with an enormous cavity at the apex; the right lung was less diseased. The heart was small, flaccid, and empty; the liver was hypertrophied. All the other organs were healthy; but the uterus was large, and its lining was of a vinous redness. M. du Saulle comments upon this phthisis as follows:—

"In the case which now occupies us, did the phthisis precede the melancholy; was it developed during the last period of lactation; or were the phthisis and the melancholy entirely mutually connected? This last opinion is ours; and we believe that the phthisis had existed and was developed in a latent state, whilst the resources of art were directed against another enemy. Our practical conclusion is, that it is very dangerous for a woman who has once been deranged, to suckle her child. Should she do so, at the period of weaning, she ought to be confided to the care of an experienced physician, who will insist upon all precautions which may prevent the return of the mental malady . . . . And as phthisis pulmonalis is a disease which appears so frequently as a sequel to prolonged lactation, and also frequently accompanies melancholia, and is developed very insidiously, it is important carefully to examine, by percussion and auscultation, all patients brought under our notice in these conditions."

We subjoin a very brief sketch of a few of the recorded cases of insanity in pregnant women. M. Baillarger relates one, of a young unmarried woman, who in the third month of her pregnancy became melancholic, apparently as the sequel to a fright, her general state having been one of depression previously. She had hallucinations, chiefly of the ear, and obstinately refused food; she had to be fed with a stomach-pump. Her mental condition gradually improved as pregnancy advanced. She went home to be confined, and shortly afterwards recovered completely. The child was healthy and strong. The same writer gives the details of another case, in which the delirium, dating from the third month, assumed the ecstatic form. She had hallucinations of both eye and ear. She recovered almost completely in about sixteen days; but, from another emotion, she had a relapse six weeks afterwards, which continued in a more or less severe form until her confinement, after which her recovery advanced rapidly, and was perfect. Both these were cases of seduction, and the moral causes appeared well marked.

Madame C——, aged 39, evinced maniacal symptoms from the period of her conception; the pregnancy advanced naturally, but the patient was always delirious. The labour was natural, and she was a little calmer after it, for a time; but the mental derangement continued incurable. She died eight years afterwards of chronic enteritis.

Dr. Reid relates an instance of melancholia, dating from conception, with continual desire to destroy the child. The mental condition became worse after delivery.

In Dr. Seymour's work on *Mental Derangement* a case is mentioned of melancholia, occurring in the fourth month of pregnancy, which was aggravated after delivery, but recovered about twelve months afterwards. M. Morel gives the history of a case, the termination of which was more serious than these. It is that of a young, hysterical, married woman, who had successive pregnancies, which aggravated the nervous state. Acute mania supervened in the seventh month of her third pregnancy; abortion took place, and the mania persisted, terminating in dementia and general paralysis.

These cases sufficiently indicate, as was before stated, that there is no constancy in the effect to be expected of labour upon the mental condition. Sometimes it is the signal for its cessation, sometimes for its aggravation; at other times it has no effect whatever.

We extract the next case, of puerperal mania proper, from M. Marcé's collection. Helen Lorig, aged 42, entered the La Charité, May 13, 1855. Her mother had had nine children; the first six confinements had been attended by no evil consequences; but after the last three she had attacks of delirium, lasting some months. H. L. was confined on the 5th of May, and gave birth to a dead child; all went on well until the 10th, when, after some moral emotion, the lochiæ were suppressed; and it was remarked that there was no evidence of lacteal secretion. There was no fever; but the ideas were slightly incoherent. On the next day the delirium became furious, and continued so till the 13th, when she was brought to the hospital. The transition produced a calmness, with which she herself was astonished. On the 14th she could answer questions for a little time, but soon began to wander again. She feared poison, and had hallucinations of the eye and ear; there was insomnia, headache, thirst, a rather dry tongue; the pulse was 100; the patient perspired freely, and the skin presented a miliary redness, with sudamina. There was a little pain on pressure over the region of the uterus. The delirium persisted, and on the 17th became almost acute: pulse 110—tr. opii, gtt. xxv. with musk, 10 grs. The same condition, and the same treatment, continued till the 21st, when there was more tranquillity, and a desire for food; the pulse was much reduced. On the 28th the pulse was 72, the uterus not painful; the patient could give a rational account of herself, and her past state, but still rambled off into other matters. On the 6th of June the delirium and hallucinations had all ceased. On the 15th she was still very weak, but rational, and left the hospital cured at the end of the month.

Our next case is from M. Esquirol:—S. J., æt. 40, entered the Salpetrière, April 22nd, 1812. Her stature is large, her face studded with pimples, her eyes and hair brown, her skin white; she is tolerably stout. History: at 12 years, headache, nasal hæmorrhage. 13 years; first appearances of the menses, cessation of the nasal hæmorrhages; since then, regular, but scanty menstruation. 18 years; a dear friend was guillotined, and she had an attack of trembling, followed by rambling, for some days. 26 years; J. married, and became the mother of three children, which she suckled. 30 years; she was confined of a fourth child, suckled it, and weaned it without precaution. Two days afterwards she was attacked with general delirium, with predominance of religious ideas. She was taken to the hospital, and left it perfectly well after four months. 36 years; a new accession, caused by the absence of her husband; she was again received into the hospital, and was there thirteen months; she left again cured. 39 years; her fifth confinement; seven months suckling; the day after weaning, delirium, with imaginary fears, broke out; she was brought into a *maison de santé* with a general eruption over the body, which, after some time, was limited to the face; twenty months after this she was received at the Salpetrière. She was sad, melancholy and despairing, and had religious terrors. On the 24th of May there was a discharge of matter from the left ear; since then she became more reasonable; she eats and sleeps well. During the month of June baths and blisters to the arms were continued; she went out in perfect health and reason on the 11th of August, 1812. All her attacks had been preluded by sadness, *ennui*, and inaptitude for her ordinary occupations; by degrees her head became confused; and during the accession she always felt her head hot and embarrassed.

M. Esquirol's cases are so instructive, from the careful manner in which the histories are traced, that we extract further. D. S. D., æt. 41, entered the Salpetrière, June 19th, 1812. Middle stature, brown hair, blue eyes, white skin, mobile physiognomy, moderate stoutness. This woman has an uncle and aunt insane. She had a fall when 9 years old, and wounded her forehead; the cicatrix is still visible. At 14 years she suffered from psora. 16 years; headache, followed by appearance of the menses, few and irregular. 26 years; D. married—from that time the menses were more abundant, with leucorrhœa; more headache. At 27 years, she had many domestic troubles during her first pregnancy. 33 years; her husband, having an operation performed without her knowledge, she was alarmed; her mind began to wander, and she became furious. She was treated at Charenton; the accession lasted five months, and the intellect remained somewhat enfeebled. She was again confined at 35 years. At 37 years, three days after her third confinement, she experienced a slight vexation, after which occurred delirium and fury. Her insanity this time lasted six months. At 41 years; domestic troubles, and slight vexations, followed by new accession of fury, lasting a few days. Received at the Salpetrière, she was calm, but not rational. August 12th; she talks much and long, relates everything that she has heard, seen or known; but with much incoherence. She has long intervals of reason. In October, she was calm, and worked; but was occasionally incoherent. In December there was the same condition. The attack terminated in dementia.

These cases, and many others which to quote would swell our paper to too great length, illustrate many important points as to the etiology of this form of insanity; and also as to the tendency to recurrence under certain conditions when the proclivity has once been developed. We must refrain from further illustration, as there are yet some important questions connected with pregnancy and labour to pass in review; and first, concerning the terminations of puerperal insanity in general—a subject which we deferred from the earlier part of the paper. We have stated that of M. Esquirol's 92 cases, 55 recovered their reason, and six died, leaving 31 incurables. Dr. Haslam reports 50 recoveries amongst 85 cases; and Dr. Burrows cured 35 out of 57. Dr. Macdonald states that 80 per cent. of his cases recovered. Dr. Brierre de Boismont says, that cases of puerperal insanity (excluding melancholia) have recovered under his care in about a week on an average—a statement which is vaguely worded, and which is open to some misunderstanding. Dr. Gooch (quoted by Dr. Pritchard) has observed that such records as these throw but little light upon the real proportion of recoveries, and present a prospect unnecessarily gloomy and discouraging; inasmuch as the "records of hospitals contain chiefly accounts of cases which have been admitted because they had been unusually permanent, having already disappointed the hope, which is generally entertained and acted upon, of relief by private care; the cases of short duration, which last only a few days or weeks, and which form a large proportion, are totally overlooked or omitted in the inspection of hospital reports." Dr. Gooch further adds—"Of the many patients about whom I have been consulted, I know only two who are now, after many years, disordered in mind; and of these one had already been so before her marriage . . . . Mania is more dangerous to life, melancholy to reason." It is extremely difficult to form an idea of the *average* duration of these cases, as will be understood from the remarks above. With regard to the prolonged cases, it is very generally received, that most of the recoveries take place within six months. In M. Esquirol's 92 cases, 55 were cured, and the recoveries took place as follows:—

| | | |
|---|---|---|
| 4 | took place in the | 1st month. |
| 7 | " | 2nd " |
| 6 | " | 3rd " |
| 7 | " | 4th " |
| 5 | " | 5th " |
| 9 | " | 6th " |
| 15 | " | in the months following. |
| 2 | " | after 2 years. |

Thus it appears that 38 out of 55, or more than two-thirds of the cures, were completed within six months from the outbreak of the mental alienation. M. Esquirol had but six deaths in the 92 cases, and none of them were fatal before the sixth month; but this must be judged by the same considerations as above stated—

viz., that the cases received at the Salpetrière were such as had passed the period generally most fatal, as will be further seen by a reference to the details already given of M. Marcé's fatal cases of mania, where all (but one) died within twenty-six days. Esquirol's fatal cases were, one after six months; one after one year; two after eighteen months; one after three years, and one after five years. Dr. Burrows' table gives 10 deaths in 57 patients, a proportion which, under ordinary circumstances, probably gives too high an average, as M. Esquirol's gives one perhaps too low.

What influence does pregnancy exert upon previously-existing mental alienation? On this question authorities differ much. M. Guislain writes as follows:—"As to the utility of conception and gestation in disorders of the intellect, opinions are much divided. I know well that these acts do not always produce the happy results that we might expect; and that delivery itself has often been the determining cause of insanity. It is, however, a truth which we ought not to doubt, that lactation almost always produces a favourable change in the moral nature of the insane." MM. Dubois and Desormeaux give this opinion:—"Mania and dementia are often favourably influenced by pregnancy; but we can only hope for a complete and durable amendment in such cases as have depended upon lesions of menstruation, or other affections of the uterus. Apart from this, pregnancy is probably unfavourable; not in itself, but by reason of the weakness induced by confinement." As in all cases connected with mental alienation so in this, M. Esquirol's opinion is of much weight:—"Pregnancy, parturition, lactation, are *sometimes* the means of which nature takes advantage to terminate attacks of alienation. I believe these terminations rare. I have seen pregnancy and parturition make the patient calmer, without affecting the delirium. I have known also a lady who became insane during five consecutive pregnancies, and each time was cured by confinement. Notwithstanding these instances, and many others cited by authors, I regard as exceptions the cases of insanity cured by marriage, pregnancy, and labour; so often have I seen the mental affection persist and even become aggravated under such circumstances. Let any one visit the Salpetrière and he will find more than 100 insane women, although they have been married and had children." A valuable collection of cases is thus summed up by M. Buchez:—"In twenty-two observations of the influence of the uterus or mammæ upon the brain affected with mental alienation, including pregnancy, labour, lactation, and weaning, *not one* has presented any diminution of the delirium; all have provoked or augmented it." M. Marcé gives a short collection of cases, from which it results that in all, at the period of pregnancy, the mental affection assumed a more serious aspect, and gave cause for suspicions of incurability.

In general, the pregnancy of the insane proceeds without any peculiarities; but there is one circumstance connected with the labour which is not only curious, but important to bear in mind Parturition is often unattended by any signs of suffering, and, in many cases, the dropping of the child upon the floor, or its cry under the bed-clothes, have been the first announcement that labour had been in progress. Such was the case in two cases noticed by M. Lannurien and M. Mitivié. A young lady attended by M. Esquirol was delivered without appearing aware of it, and also without any of the attendants knowing. Many cases of similar import are related by M. Marcé, who considers that painless labour is the rule in confirmed cases of insanity. And thus it appears that the analgesia, which is so frequent an accompaniment of mental deragement, may extend to the internal organs. Nor is this an isolated fact in the history of insanity; it is borne out by the phenomena of phthisis, pneumonia, pleurisy, and other diseases, occurring in such a state, which frequently, or even generally, occur without the usual constitutional or physical signs. It results from this that the insane, near the period of their confinement, require to be watched and guarded with especial care.

The children born of insane mothers are naturally submitted to the most unfavourable influences, and can but rarely escape the hereditary taint—here in tenfold force. Those born of mothers who have become insane during their pregnancy are not unfrequently still-born; M. Marcé states that of 11 such cases five were still-born. Those born of mothers insane before their pregnancy are not found to be dead in so large a proportion of cases. The intellectual condition of those who survive and grow up is generally most lamentable; they remain imbecile, or at least of a very limited intelligence. There are, however, instances in which the child has betrayed no trace of its disordered source. Catherine, the daughter of Jeanne la Folle, was born during her mother's insanity, and afterwards became Queen of Portugal, and presented no indications of unsound mind. M. Marcé has collected a few cases in which similar immunity was manifested; some from his own note-book, and two from those of MM. Calmeil and Baillarger; but these must be looked upon as only exceptional: the future of a child born under such inauspicious circumstances must be the subject of apprehension of the very gravest character.

We intended to have appended some observations upon the legal responsibility of pregnant women; but the subject is one of too much importance, and too much contested, to be entered upon in the brief limits which are still at our disposal.

